# Occurrence rate and species and subtypes of *Cryptosporidium* spp. in pet dogs in Yunnan Province, China

**DOI:** 10.1186/s12866-024-03500-4

**Published:** 2024-09-19

**Authors:** Jinhua Jian, Aiqin Liu, Yaming Yang, Xiaoxue Peng, Lan Yao, Benfu Li, Jinrong Zi, Jianping Cao, Yujuan Shen

**Affiliations:** 1 National Key Laboratory of Intelligent Tracking and Forecasting for Infectious Diseases, National Institute of Parasitic Diseases, Chinese Center for Disease Control and Prevention (Chinese Center for Tropical Diseases Research), NHC Key Laboratory of Parasite and Vector Biology, National Center for International Research on Tropical Diseases, Shanghai, 200025 China; 2 School of Basic Medical Sciences and Forensic Medicine, Hangzhou Medical College, Hangzhou, Zhejiang 310000 China; 3 Department of Parasitology, Harbin Medical University, Harbin, 150081 China; 4 Department of Helminth, Yunnan Institute of Parasitic Diseases, Puer, 655099 China

**Keywords:** *Cryptosporidium*, Genotyping, Subtyping, Dogs, Zoonotic transmission

## Abstract

**Background:**

*Cryptosporidium* spp. is a ubiquitous, globally distributed intestinal protozoan infecting humans and at least 260 animal hosts. Due to close human contact with pet dogs and identification of zoonotic *Cryptosporidium* species and subtypes in these animals, dog health is not only a veterinarian issue but also a public health issue. This study aimed to understand occurrence and genetic characterization at both genotype and subtype levels in pet dogs in Yunnan Province, China.

**Results:**

A total of 589 fresh fecal specimens were collected from adult pet dogs in the rural areas of eight cities/autonomous prefectures of Yunnan Province, China. 16 fecal specimens were positive for *Cryptosporidium* spp. by polymerase chain reaction (PCR) amplification and sequence analysis of the small subunit ribosomal RNA (*SSU rRNA*) gene, with an average occurrence rate of 2.7% (16/589) being observed. Three zoonotic *Cryptosporidium* species were identified: *C. parvum* (*n* = 7), *C. suis* (*n* = 5) and *C. canis* (*n* = 4). At the 60-kDa glycoprotein (*gp60*) locus, only three *C. parvum* and two *C. canis* specimens were successfully amplified and sequenced, with subtype IIaA17G2R1 (*n* = 3) and subtypes XXa4 (*n* = 1) and XXa5 (*n* = 1) being identified, respectively.

**Conclusions:**

The present finding of three zoonotic *Cryptosporidium* species in dogs implied that dogs infected with *Cryptosporidium* spp. may pose a threat to human health. *C. suis* was identified in dogs in this study for the first time, expanding the host range of this species. Identification of *C. parvum* subtype IIaA17G2R1 and *C. canis* subtypes XXa4 and XXa5 will be helpful to explore the source attribution of infection/contamination and assess the transmission dynamics of *C. parvum* and *C. canis* in the investigated areas in the future.

## Background

 Pets are widely recognized as an integral part of modern society worldwide. Pet dogs are the most common companion animals, with the number of pet dogs increasing in recent years. The population of pet dogs was estimated to be between 83.7 million and 88.9 million in 2020, up 9.0–16.0% from year-end 2016 (https://avmajournals.avma.org/display/post/news/pet-population-still-on-the-rise--with-fewer-pets-per-household.xml). However, dogs have been reported to be reservoirs, carriers, and transmitters of some parasites, including zoonotic intestinal protozoan *Cryptosporidium* spp. [1]. Since the first report of this parasite in dogs in 1981, more than 100 publications have documented *Cryptosporidium* infections in dogs from 27 different countries worldwide [2, 3].

*Cryptosporidium* spp. is a ubiquitous, globally distributed intestinal protozoan infecting humans and animals. Infectious *Cryptosporidium* oocysts are excreted with host feces into environments. Humans can acquire *Cryptosporidium* infections through the fecal–oral route, and four modes of transmission have been demonstrated: person-to-person transmission, animal-to-person transmission, foodborne transmission, and waterborne transmission [4]. Due to the wide animal host range of *Cryptosporidium* spp. (at least 260 species), the epidemiological role animals play in the transmission of human cryptosporidiosis has been receiving more concern [5]. Many studies have indicated that contact with animals is considered a risk factor for human *Cryptosporidium* infection [6]. In fact, to date, more than 20 outbreaks of cryptosporidiosis related to contact with animals have been reported [7]. Recent two-decade molecular epidemiological studies documented occurrence (average rate: 5.3% (929/17409)) of *Cryptosporidium* spp. in dogs, and zoonotic *C. canis* and *C. parvum* are the two most common species in these animals (Table 1). The findings above have raised awareness of zoonotic transmission potential of cryptosporidiosis due to contact with dogs.

**Table 1:** Prevalence of *Cryptosporidium* spp. and distribution of species and genotypes/subtypes in dogs worldwide

Source	Country	Positive no./Examined no. (%)	Species and genotypes (n)/Subtypes (n)	Reference
Shelter	China	43/871 (4.9)	*C. canis* (35); *C. parvum* (7); *C. ubiquitum* (1)	[8–13]
	Egypt	6/220 (2.7)	*C. canis* (6)	[14]
	Greece	41/278 (14.7)^a^	*C. scrofarum* (1)	[15]
	Italy	3/285 (1.1)	*C. parvum* (3)	[16]
	Japan	13/140 (9.3)	*C. canis* (13)	[17]
	Poland	2/101 (2.0)	*C. canis* (1)/XXe1 (1); *C. proliferans* (1)	[18]
	Spain	8/194 (4.1)^a^	*C. canis* (5); *C. hominis* (1)	[19]
	Thailand	4/540 (0.7)	*C. canis* (4)	[20]
	UK	31/676 (4.6)	*C. canis* (28); *C. parvum* (2)/IIaA17G1R1 (1), IIaA15G2R1 (1); *C. andersoni* (1)	[21]
	Subtotal	151/3305 (4.6)	*C. canis* (92)/XXe1 (1);* C. parvum* (12)/ IIaA17G1R1 (1), IIaA15G2R1 (1); *C. hominis* (1); *C. ubiquitum *(1);* C. andersoni *(1); *C. scrofarum* (1); *C. proliferans* (1)	
Breeding center	China	37/1009 (3.7)	*C. canis* (36); *C. parvum* (1)	[8, 9, 22–24]
	Germany	35/349 (10.0)	*C. canis* (33)/XXd1 (5), XXe1 (6), XXb1 (1), XXb2 (1), XXb3 (1); *C. parvum *(2)/IIaA15G2R1 (2)	[25]
	Italy	7/759 (0.9)	*C. parvum* (6); *C. canis* (1)	[26, 27]
	Japan	66/314 (21.0)	*C. canis* (66)	[28]
	USA	49/70 (70.0)^a^	*C. muris* (6)	[29]
	Subtotal	194/2501 (7.8)	*C. canis* (136)/ XXd1 (5), XXe1 (6), XXb1 (1), XXb2 (1), XXb3 (1); *C. parvum *(9)/IIaA15G2R1 (2); *C. muris* (6)	
Pet market	China	64/1099 (5.8)	*C. canis* (63); *C. muris* (1)	[8, 13, 22–24, 30]
	Japan	149/471 (31.6)^a^	*C. canis* (75)	[31]
	Subtotal	213/1570 (13.6)	*C. canis *(138); *C. muris* (1)	
Farm	China	4/81 (4.9)	*C. canis* (4)	[32]
	Iran	2/140 (1.4)	*C. parvum* (2)	[33]
	Subtotal	6/221 (2.7)	*C. canis *(4); *C. parvum *(2)	
Pet/Household	Brazil	16/197 (8.1)^b^	*C. canis* (5); *C. parvum* (3)/IIaA17G2R2 (2)	[34, 35]
	Canada	21/860 (2.4)^a^	*C. canis* (3)	[36]
	China	16/398 (4.0)	*C. canis* (15); *C. parvum* (1)	[8, 9, 12]
	Egypt	25/348 (7.2)	*C. canis* (13)/XXe2 (1); *C. parvum* (5);* C. *spp. (7)	[14, 37, 38]
	France	3/116 (2.6)	*C. canis* (3)	[39]
	Greece	11/601 (1.8)^a^	*C. canis* (2)	[15]
	Italy	2/120 (1.7)	*C. parvum* (2)	[27]
	Japan	41/606 (6.8)^a^	*C. canis* (29)	[31, 40]
	Poland	9/264 (3.4)	*C. canis* (3); *C. parvum* (2); *C. *spp. (4)	[41]
	USA	2/128 (1.6)^a^	*C. canis* (1)	[42]
	Vietnam	1/3 (33.3)	*C. canis* (1)	[43]
	Subtotal	147/3641 (4.0)	*C. canis *(75)/XXe2 (1); *C. parvum *(13) /IIaA17G2R2 (2); *C. *spp. (11)	
Clinic	Brazil	6/128 (4.7)	*C. canis* (4); *C. parvum* (2)	[44]
	China	61/1896 (3.2)	*C. canis* (56); rat genotype IV (1); *C.* spp. (4)	[8, 10, 13, 22, 24, 30, 45–47]
	Germany	1/81 (1.2)	*C. parvum* (1)	[48]
	Iran	2/315 (0.6)	*C. canis* (2)	[49]
	Japan	18/98 (18.4)^a^	*C. canis* (10)	[31]
	Spain	5/252 (2.0)	*C. canis* (4)/XXi1 (1); *C. parvum* (1)	[50]
	USA	6/84 (7.1)^a^	*C. canis* (4)	[51]
	Subtotal	99/2854 (3.5)	*C. canis *(80)/XXi1 (1);* C. parvum *(4); rat genotype IV (1); *C. *spp. (4)	
Others^c^	Australia	8/1400 (0.6)^a^	*C. canis* (4)	[52]
	Canada	13/140 (9.3)^a^	*C. canis* (4)	[53]
	China	30/932 (3.2)	*C. canis* (27); *C. meleagridis* (1); *C.* spp. (2)	[54, 55]
	Czech Republic	2^d^	*C. parvum* (1); *C. meleagridis* (1)	[56]
	Italy	11/435 (2.5)	*C. parvum* (10); *C. canis* (1)	[57]
	Thailand	57/410 (13.9)^a^	*C. canis* (23); *C. parvum* (8); *C. canis*+*C. parvum* (1)	[58, 59]
Total		929/17409 (5.3)	*C. canis* (584)/XXe1 (7), XXd1 (5), XXb1 (1), XXb2 (1), XXb3 (1), XXe2 (1), XXi1 (1);* C. parvum *(58)/IIaA15G2R1 (3), IIaA17G1R1 (1), IIaA17G2R2 (2);* C. muris* (7);* C. hominis* (1);* C. meleagridis* (1); *C. andersoni* (1); *C. scrofarum *(1); rat genotype IV (1);* C. ubiquitum* (1);* C. proliferans *(1);* C. canis*+*C. parvum* (1); *C. *spp.(17)	

Note: *C.* spp. indicated that *Cryptosporidium* spp. were untyped

^a^The prevalences of *Cryptosporidium* spp. were determined by morphological or immunological methods. Only some of *Cryptosporidium*-positive specimens were successfully genotyped by PCR and sequencing

^b^Of the 11 infected dogs, only specimens from three dogs living with infected children were subjected to molecular analysis

^c^Others contained the dogs not having the specific number of specimens related to the sources or having unclear sources

^d^Only *Cryptosporidium*-positive specimens were genotyped in the study conducted in the Czech Republic

Within the genus *Cryptosporidium*, extensive genetic variation has been confirmed. Currently, PCR-based molecular techniques targeting the small subunit of ribosomal RNA (*SSU rRNA*) gene have been widely used in the identification of *Cryptosporidium* species/genotypes. To date, at least 49 valid *Cryptosporidium* species and more than 120 genotypes have been described, with 23 species and two genotypes being identified in both humans and animals [60–62]. In dogs, nine *Cryptosporidium* species (*C. canis*, *C. parvum*, *C. muris*, *C. hominis*, *C. ubiquitum*, *C. meleagridis*, *C. andersoni*, *C. scrofarum* and *C. proliferans*) and one genotype (*Cryptosporidium* rat genotype IV) have been reported (Table 1). *C. canis s*howed an absolute predominance in dogs, accounting for 86.8% (585/674) of dog-derived *Cryptosporidium* isolates (Table 1). Since more than 100 confirmed human *C. canis* infections have been reported, the public health problem that dogs cause as a reservoir host of *Cryptosporidium* spp. should be paid more attention [63]. A recently developed *gp60* subtyping tool for *C. canis* has greatly improved our understanding of the transmission of this species [63]. The observation of three *C. canis* subtypes (XXa1, XXa4 and XXb2) identified in both dogs and humans supports the potential of zoonotic transmission of *C. canis* from dogs to humans [25, 63].

In China, since the first report of *Cryptosporidium* spp. in dogs in 2014, 16 molecular epidemiological studies of *Cryptosporidium* spp. have been carried out in dogs, and overall occurrence rate was 4.1% (255/6286) with five species (*C. canis*, *C. parvum*, *C. meleagridis*, *C. ubiquitum* and *C. muris*) and one genotype (rat genotype IV) being identified (Table 1). In southern China, especially in rural areas where ethnic minorities congregate, keeping dogs nearly becomes a common habit of inhabitants under the influence of local customs and cultures. The dogs generally serve well as protectors and companions. The aim of this study was to determine occurrence rate of *Cryptosporidium* spp. and distribution of species and genotypes/subtypes in pet dogs from the rural areas of eight cities/autonomous prefectures of Yunnan Province, China. Meanwhile, the potential of zoonotic transmission and the significance of public health of dog-derived *Cryptosporidium* isolates were also assessed by analyzing their genetic characterization at both genotype and subtype levels.

## Results

### Occurrence rates of *Cryptosporidium* spp. in pet dogs

A total of 16 (2.7%) of 589 fecal specimens were determined to be infected with *Cryptosporidium* spp. by PCR amplification and sequence analysis of the *SSU rRNA* gene. *Cryptosporidium* spp. was found in pet dogs in six autonomous prefectures/cities. The occurrence rates were 6.0% in Zhaotong, 3.7% in Kunming, 3.4% in Dali Bai, 3.4% in Nujiang Lisu, 2.0% in Lijiang and 0.7% in Diqing Tibetan. There was an absence of *Cryptosporidium* spp. in pet dogs in Chuxiong Yi Autonomous Prefecture and Baoshan City of Yunnan Province (Table 2).

**Table 2:** Occurrence of *Cryptosporidium* species and subtypes in pet dogs in Yunnan Province, China

Sampling site (N, E)^a^		Examined no.	Positive no. (%)	Species (n)	Subtypes (n)
Autonomous Prefecture	Chuxiong Yi (24°13′─26°30′N, 100°43′─102°32′E)	11	0		
	Dali Bai (24°41′─26°42′N, 98°52′─101°03′E)	59	2 (3.4)	*C. parvum* (1), *C. sui*s (1)	IIaA17G2R1 (1)
	Nujiang Lisu (25°33′─28°23′N, 98°39′─99°39′E)	204	7 (3.4)	*C. parvum* (4), *C. suis* (3)	IIaA17G2R1 (2)
	Diqing Tibetan (26°52′─29°16′N, 98°20′─100°19′E)	151	1 (0.7)	*C. canis* (1)	
City	Baoshan (24°08′─25°51′N, 98°25′─100°02′E)	10	0		
	Kunming (24°23′─26°33′N, 102°10′─103°40′E)	54	2 (3.7)	*C. canis* (1), *C. suis* (1)	XXa4 (1)
	Lijiang (25°59′─27°55′N, 99°22′─101°30′E)	50	1 (2.0)	*C. parvum* (1)	
	Zhaotong (26°55′─28°36′N, 102°52′─105°19′E)	50	3 (6.0)	*C. canis* (2), *C. parvum* (1)	XXa5 (1)
Total		589	16 (2.7)	*C. parvum* (7), *C. suis* (5), *C. canis* (4)	IIaA17G2R1 (3), XXa4 (1), XXa5 (1)

^a^N and E indicate north latitude and east longitude, respectively

### Molecular characteristics of *Cryptosporidium* spp. at the *SSU rRNA *Locus

Of the 16 *Cryptosporidium*-positive specimens, three *Cryptosporidium* species were identified: *C. parvum* (*n* = 7), *C. suis* (*n* = 5) and *C. canis* (*n* = 4). *C. suis* was found in dogs for the first time. *C. parvum* and *C. suis* showed genetic diversity at the *SSU rRNA* gene locus. Five different *C. parvum* sequences (accession number: PP896999–PP897003) were obtained with six polymorphic sites being observed. Five different *C. suis* sequences (accession number: PP897004–PP897008) were found with nine polymorphic sites being observed. In contrast, four obtained *C. canis* sequences were identical to each other (accession number: PP896998).

### Molecular characteristics of *Cryptosporidium* spp. at the *gp60* Locus

Seven *C. parvum* and four *C. canis* DNA specimens were further subtyped by PCR amplification and sequence analysis of the *gp60* gene. However, only three *C. parvum* and two *C. canis* specimens produced the expected PCR amplicons and were successfully sequenced. Three *gp60* gene sequences of *C. parvum* were identical to each other (accession number: PP887783) and had 100% similarity with the sequence of subtype IIaA17G2R1 (accession number: PP112337). Two *gp60* gene sequences of *C. canis* (accession number: PP887781–PP887782) had two base differences. They had the largest similarity (100% and 99.7%) with the sequence of subtype XXa4 (accession number: MT954607) and two subtypes of *C. canis* were identified: XXa4 and XXa5.

## Discussion

Dogs have been reported to be infected naturally with zoonotic *Cryptosporidium* spp. Thus, the detection of *Cryptosporidium* spp. in dogs is not only a veterinarian issue but also a public health issue. In this study, the occurrence rate of *Cryptosporidium* spp. was 2.7% in the investigated dogs, which was lower than the pooled rate in dogs worldwide (5.3%) (Table 1). In fact, the occurrence rates of *Cryptosporidium* spp. are complicated in animals and probably affected by many factors, including age and health status of animals and their living conditions, and so on. *Cryptosporidium* spp. is considered as a causative agent of opportunistic infection. Health status of hosts is the key factor for its infection, and it is closely associated with host ages. Currently, a lot of studies on the relationship between the occurrence rates of *Cryptosporidium* spp. and the age of dogs indicated that *Cryptosporidium* infections are the most common in puppies (< 1-year-old dogs) [8, 20, 28, 52, 59, 64, 65]. This finding may be related to the immature immune system of young animals. In a meta-analysis of the global prevalence of *Cryptosporidium* infection in dogs, a positive relationship was also observed between *Cryptosporidium* infection and diarrhea in dogs [3]. A recent study demonstrated that the risk of being positive for *Cryptosporidium* spp. was 2.9-fold higher in the diarrheal dogs than in the no-obvious-clinical-sign dogs [55]. The occurrence rate of *Cryptosporidium* spp. was significantly higher in diarrheal dogs than in non-diarrheal ones in two studies: 30.0% vs. 4.2% in Northeast Spain [66]; 30.3% vs. 16.9% in Nigeria [67]. On the contrary, a higher but non-significant prevalence of *Cryptosporidium* spp. was found in non-diarrheic dogs than in diarrheic dogs in Thailand (32.6% vs. 23.5%) [58]. The inconsistent conclusion may be related to the number of infecting *Cryptosporidium* oocysts. Additionally, living conditions of dogs might have influence on *Cryptosporidium* infection [68]. Based on current dog epidemiological data of *Cryptosporidium* spp., it was observed that dogs in pet markets had the highest pooled occurrence rate (13.6%, 213/1570) (Table 1). Usually, the dogs in pet markets have the limited living spaces, leading to close contact with each other. Meanwhile, insufficient sanitary control of facilities easily induces transmission of *Cryptosporidium* spp.. Recently, there were two studies revealing that outdoor dogs had a higher prevalence of *Cryptosporidium* infection than indoor dogs conducted in Nigeria (26.1% vs. 14.7%) and Iran (79.4% vs. 20.6%) [49, 69]. In fact, early in 2007, Rambozzi et al., reported that outdoor dogs were approximately five times more likely to be infected with *Cryptosporidium* spp. than indoor ones [70]. This phenomenon can be attributed to the fact that unconfined/non-sheltered dogs can roam about in the streets for hunting, foraging and eating adulterated food materials. This increases the risk of getting infected with *Cryptosporidium* spp. In this study, all the dogs were adult and had no clinical signs of illness at the time of sampling. Meanwhile, all the dogs are from rural areas, and often kept in the courtyard. The limited range of dog activities might be a relatively low risk of *Cryptosporidium* infection for these animals.

In this study, sequence analysis of *Cryptosporidium* spp. at the *SSU rRNA* locus identified three zoonotic *Cryptosporidium* species (*C. parvum*, *C. canis* and *C. suis*), suggesting the potential of dog-related zoonotic transmission of *Cryptosporidium* spp. in the investigated areas.

*C. parvum* is often found in dogs worldwide, and sometimes showed predominance in these animals. In a recent study of molecular detection of *Cryptosporidium* spp. in dog fecal specimens contaminating public areas in Northern Italy, *C. parvum* accounted for > 90.0% of *Cryptosporidium*-positive specimens [57]. In fact, *C. parvum* is one of the two most common *Cryptosporidium* species causing human cryptosporidiosis [71]. Epidemiological studies have identified *C. parvum* in more than 155 mammalian species [5]. Thus, *C. parvum* is of concern as a major zoonotic *Cryptosporidium* species. In this study, *C. parvum* subtype IIaA17G2R1 was identified in dogs for the first time. In fact, molecular epidemiological studies of *Cryptosporidium* spp., revealed that subtype IIaA17G2R1 was relatively common in livestock, occurring mainly in cattle [72] but appearing occasionally in other animals including sheep, camels, goats, rodents, mustangs and fish [73–78]. This subtype has also been reported in human cases of cryptosporidiosis in Iran and Jordan [79, 80]. In North Carolina of the US in 2009, there was an outbreak of human cryptosporidiosis caused by subtype IIaA17G2R1 in a youth summer camp. Meanwhile, this subtype was identified in both humans and livestock in the camp setting, indicating that zoonotic transmission had occurred [81]. Our finding of subtype IIaA17G2R1 in dogs suggests necessity to take measures to reduce the risk of zoonotic transmission of *Cryptosporidium* spp. from dogs to humans.

*C. canis* is the most often identified species in dogs and is considered a host-adapted species in dogs [68]. The global epidemiologic data revealed that 86.8% (585/674) of dog-derived C*ryptosporidium* isolates belonged to *C. canis* (Table 1). *C. canis* was also found in some other animals, including foxes, coyotes, minks, mongooses, raccoon dogs, cattle and sheep as well as rodent animals [82–84]. *C. canis* is one of common zoonotic *Cryptosporidium* species. However, most human infections with *C. canis* were reported in people in developing countries or those who have traveled to developing countries [63]. *C. canis* was responsible for as much as 4.0% of overall cryptosporidiosis cases in children in developing countries [85]. With the increasing case number of human cryptosporidiosis caused by *C. canis* (more than 100 cases worldwide), the transmission characteristics and public health significance of *C. canis* need to be studied extensively and deeply [63]. A recently developed *gp60* subtyping tool for *C. canis* can improve our understanding of the transmission of this zoonotic species. In this study, *C. canis* isolates were further subtyped, and a known subtype XXa4 and a novel subtype XXa5 were identified. Subtype XXa4 of *C. canis* has been reported in dogs, foxes and raccoon dogs and humans from Ethiopia and Peru (Table 3). To date, according to *gp60* subtyping data of *C. canis*, a total of 20 subtypes belonging to nine subtype families (XXa–XXi) have been identified including subtype XXa5 newly identified in this study [50], and 11 subtypes (XXa1, XXa4, XXa5, XXb1–XXb3, XXd1, XXe1, XXe2, XXh1 and XXi1) could be seen in dogs (Table 3). Six subtypes (XXa1–XXa4, XXb2 and XXc1) have been reported in humans, with four subtypes being identified in both humans and animals: XXa1, XXa4 and XXb2 in dogs, XXa2 and XXa4 in raccoon dogs and XXa4 in foxes (Table 3), indicating the zoonotic potential of these subtypes. In fact, the occurrence of zoonotic transmission of cryptosporidiosis caused by subtype XXa4 has been confirmed based on the fact that the children and their dogs in a household belonged to subtype XXa4 [63, 86].

**Table 3:** Host and geographical distribution of *gp60* subtypes of *Cryptosporidium* *canis*

Host	Subtype family	Subtypes (country)	Reference
Human	XXa	XXa1 (Peru); XXa2 (Jamaica); XXa3 (Peru, Kenya); XXa4 (Peru, Ethiopia)	[63]
	XXb	XXb2 (China)	[63]
	XXc	XXc1 (Kenya)	[63]
Dog	XXa	XXa1 (Peru); XXa4 (Peru, USA); **XXa5 (China)**	[63]; This study
	XXb	XXb1 (China, Germany); XXb2(Germany); XXb3 (Germany)	[25, 63]
	XXd	XXd1 (USA, Germany)	[25, 63]
	XXe	XXe1 (USA, Poland, Germany); XXe2 (Egypt)	[18, 25, 38, 63]
	XXh	XXh1 (Germany)	[50]
	XXi	XXi1 (Spain)	[50]
Raccoon dog	XXa	XXa2 (China); XXa4 (China)	[87]
Fox	XXa	XXa4 (China)	[88]
	XXb	XXb3 (USA)	[63]
	XXg	XXg1 (China); XXg2 (China); XXg3 (China)	[87]
Mink	XXd	XXd2 (China)	[87]
	XXf	XXf1 (China); XXf2 (China)	[87]

Note: Bold letters indicate the newly discovered subtype in this study

To our knowledge, *C. suis* was reported in dogs for the first time in this study. It is well-known that *C. suis* is the dominant species infecting pigs (especially in pre-weaned pigs) [89]. The accidental finding of *C. suis* in dogs might be closely related to the fact that coprophagia is a frequent practice in these animals. In the investigated areas, almost all the families raised pigs for consumption. Combined with the observation of *C. suis* mostly found in pigs previously [90], pigs were suspected to be the source of *C. suis* infection in dogs in the investigated areas. Dogs could acquire *C. suis* infections by exposure to fecal materials from pigs in their environment. *C. suis* was also found in other animals, such as cattle, red deer and rodent animals [91–93]. Human cases of cryptosporidiosis caused by *C. suis* are occasionally reported in some countries, including China, Thailand, Cambodia, Peru, the UK and Madagascar [91, 94–98]. Currently, we could not confirm actual infections or mechanical transmission of *C. suis* in dogs only based on molecular detection of *C. suis* in dog fecal specimens. The epidemiological role of dogs in the transmission of cryptosporidiosis caused by *C. suis* needed to be assessed.

## Conclusions

In conclusion, this study described the occurrence (2.7%, 16/589) and genetic characterization of *Cryptosporidium* spp. at both genotype and subtype levels in pet dogs in Yunnan Province, China. The finding of three zoonotic *Cryptosporidium* species (*C. parvum*, *C. canis* and *C. suis*) in dogs implied that dogs infected with *Cryptosporidium* spp. may pose a threat to human health. High frequency of human contact with pet dogs increases the risk of human *Cryptosporidium* infection. Therefore, health education to reduce dog-related zoonotic transmission of *Cryptosporidium* infection should be carried out among people, especially pet owners and veterinarians having close contact with these animals. *C. suis* was identified in dogs in this study for the first time, expanding the host range of this species. Identification of *C. parvum* subtype IIaA17G2R1 and *C. canis* subtypes XXa4 and XXa5 will be helpful to explore the source attribution of infection/contamination and assess the transmission dynamics of *C. parvum* and *C. canis* in the investigated areas in the future.

## Methods

### Fecal specimen collection

During April to June 2021, 589 fecal specimens (each approximately 15–20 g) were collected from adult pet dogs from the rural areas of eight cities/autonomous prefectures of Yunnan Province, China. Detailed sampling sites and specimen numbers were shown in Table 2. One fecal specimen of each animal was taken immediately from fresh feces on the ground after defecation using a sterile disposable latex glove and was then placed into plastic zippered bags individually. Meanwhile, each specimen was given a unique identification number. No clinical signs of diarrhea were observed in any of the animals at the time of specimen collection. All the collected specimens were transported to our laboratory in a cooler with ice packs and were stored at − 20 °C in a freezer.

### DNA extraction

Genomic DNA was extracted from approximately 180–200 mg of each specimen using a QIAamp DNA Stool Mini Kit (QIAgen, Hilden, Germany), and the procedures and reagents utilized were provided by the manufacturer. The lysis temperature was increased to 95 °C due to difficulty in lysing *Cryptosporidium* oocysts. The extracted DNA (200 µl) was stored at − 20 °C until being analyzed by PCR.

### *Cryptosporidium* genotyping and subtyping

The partial small subunit (*SSU*) *rRNA* gene (approximately 830 bp) was amplified by nested PCR to identify *Cryptosporidium* species and genotypes. The primers and the cycling parameters were used as described previously by Huang et al. [99]. The *C. parvum*- and *C. canis*-positive specimens were analyzed to determine subtypes by nested PCR amplification of the *gp60* gene (approximately 400 bp for *C. parvum* and 700 bp for *C. canis*). The primers and the cycling parameters were used as described previously by Sulaiman et al. for *C. parvum* [100] and by Jiang et al. for *C. canis* [63]. Two replicates were used in PCR analysis of each specimen at each locus to increase detection rate, with both negative and positive controls being included in each PCR test. All secondary PCR products were subjected to electrophoresis in 1.5% agarose gels and visualized by staining the gel with GelStrain (TransGen Biotech., Beijing, China).

### Nucleotide sequencing and analyzing

For each target gene, all positive secondary PCR products of expected size were sequenced in both directions on an ABI PRISM 3730XL DNA Analyzer by Comate Bioscience Company Limited (Jilin, China), using the BigDye Terminator v3.1 Cycle Sequencing Kit (Applied Biosystems, Carlsbad, CA, USA). All raw sequencing data obtained in the present study were assembled using the software MEGA 11.0 (https://www.megasofware.net/). Edited nucleotide sequences were subjected to BLAST searches (http://www.ncbi.nlm.nih.gov/blast/) to determine *Cryptosporidium* species and subtypes.

## Data Availability

The representative nucleotide sequences obtained in the present study were deposited in GenBank database under the following accession nos.: PP896998–PP897008 (*SSU rRNA*), PP887781–PP887783 (*gp60*).

## Abbreviations

PCR: Polymerase chain reaction
*SSU rRNA*: Small subunit ribosomal RNA
*gp60*: 60-kDa glycoprotein
